# The role of facemasks and hand hygiene in the prevention of influenza transmission in households: results from a cluster randomised trial; Berlin, Germany, 2009-2011

**DOI:** 10.1186/1471-2334-12-26

**Published:** 2012-01-26

**Authors:** Thorsten Suess, Cornelius Remschmidt, Susanne B Schink, Brunhilde Schweiger, Andreas Nitsche, Kati Schroeder, Joerg Doellinger, Jeanette Milde, Walter Haas, Irina Koehler, Gérard Krause, Udo Buchholz

**Affiliations:** 1 Department of Infectious Disease Epidemiology, Robert Koch Institute, DGZ-Ring 1, 13086 Berlin, Germany; 2 National Reference Centre for Influenza, Robert Koch Institute, Berlin, Germany; 3 Centre for Biological Security, Division of Highly-Pathogenic Viruses (ZBS1), Robert Koch Institute, Berlin, Germany

## Abstract

**Background:**

Previous controlled studies on the effect of non-pharmaceutical interventions (NPI) - namely the use of facemasks and intensified hand hygiene - in preventing household transmission of influenza have not produced definitive results. We aimed to investigate efficacy, acceptability, and tolerability of NPI in households with influenza index patients.

**Methods:**

We conducted a cluster randomized controlled trial during the pandemic season 2009/10 and the ensuing influenza season 2010/11. We included households with an influenza positive index case in the absence of further respiratory illness within the preceding 14 days. Study arms were wearing a facemask and practicing intensified hand hygiene (MH group), wearing facemasks only (M group) and none of the two (control group). Main outcome measure was laboratory confirmed influenza infection in a household contact. We used daily questionnaires to examine adherence and tolerability of the interventions.

**Results:**

We recruited 84 households (30 control, 26 M and 28 MH households) with 82, 69 and 67 household contacts, respectively. In 2009/10 all 41 index cases had a influenza A (H1N1) pdm09 infection, in 2010/11 24 had an A (H1N1) pdm09 and 20 had a B infection. The total secondary attack rate was 16% (35/218). In intention-to-treat analysis there was no statistically significant effect of the M and MH interventions on secondary infections. When analysing only households where intervention was implemented within 36 h after symptom onset of the index case, secondary infection in the pooled M and MH groups was significantly lower compared to the control group (adjusted odds ratio 0.16, 95% CI, 0.03-0.92). In a per-protocol analysis odds ratios were significantly reduced among participants of the M group (adjusted odds ratio, 0.30, 95% CI, 0.10-0.94). With the exception of MH index cases in 2010/11 adherence was good for adults and children, contacts and index cases.

**Conclusions:**

Results suggest that household transmission of influenza can be reduced by the use of NPI, such as facemasks and intensified hand hygiene, when implemented early and used diligently. Concerns about acceptability and tolerability of the interventions should not be a reason against their recommendation.

**Trial registration:**

The study was registered with ClinicalTrials.gov (Identifier NCT00833885).

## Background

Since 2006, the World Health Organisation (WHO) and other organisations have highlighted the need for controlled trials to assist in formulating recommendations on the use of non-pharmaceutical interventions (NPI) - such as facemasks or hand hygiene measures - as options to prevent influenza transmission, particularly in households [1,2]. As these measures are easily applicable and accessible for the general population and do not rely on microbiological data they could be available even in very early stages of a pandemic. Several controlled trials testing NPI have lately been undertaken in different settings and with different designs [3-7] but have not been able to provide conclusive results. Four of these studies were conducted within households [4-7], one among adults living in university residence halls [3]. All but one [7] took place during seasonal influenza epidemics. Regarding interventions, one study compared facemasks to respirators [6], another evaluated facemasks only [4], while three studies assessed facemasks, and/or hand hygiene measures in various combinations [3,5,7].

In intention-to-treat analysis, none of the four household based trials was able to show significant reductions in secondary attack rates (SAR) when comparing intervention to control groups. However, one subgroup-analysis (restricting intervention households to those where intervention started within 36 h of symptom onset) [5] and one per-protocol analysis [6] showed significant effects of the interventions. Similarly, cumulative incidence in the study among university students yielded no significant differences between study arms, but survival analysis identified significant reductions of influenza-like-illness (ILI) in weeks 4-6 after recruitment [3].

In all publications, it was hypothesized that the effect of interventions may be more pronounced in the case of an influenza pandemic, due to higher public anxiety with resulting higher rates of adherence. This was supported by observations made in a comparable crisis, namely the SARS epidemic [8].

During the pandemic of influenza A (H1N1)pdm09 there was a considerable amount of uncertainty among public health officials if or which NPI should be recommended. Germany, in accordance with many other countries, did not encourage the widespread use of facemasks; hand washing had been generally recommended against the transmission of respiratory viruses already before the pandemic. Further evidence based data on NPI from controlled studies are still needed, as they are necessary to inform decision makers on the potential benefit of NPI in influenza pandemics.

Between 2009 and 2011 we conducted a cluster randomised trial on the efficacy, adherence and tolerability of facemasks and intensified hand hygiene to prevent influenza transmission in households. Study results from the 2009/2010 pandemic season concerning data about adherence and tolerability only have already been published [9].

## Methods

### Design

We conducted the study during two consecutive influenza seasons (November 2009-January 2010 and January-April 2011) in Berlin, Germany. Index patients were recruited by general practitioners or pediatricians. We cooperated with 20 study sites in 2009/10 and 12 study sites in 2010/11 evenly distributed in the city of Berlin. We included index patients if they presented at the study sites within 2 days of symptom onset, had a positive rapid antigen test for influenza (later to be confirmed by quantitative Reverse Transcription Polymerase Chain Reaction [qRT-PCR]), and were at least 2 years old. Index cases also had to be the only household member suffering from respiratory disease within 14 days prior to symptom onset. Exclusion criteria were pregnancy, severely reduced health status and HIV infection. One person households were also not eligible for inclusion.

### Informed consent

We obtained written informed consent from all study participants. If these were less than 18 years of age we asked their parents or legal guardians to provide proxy written consent, with additional written consent from those participants aged 14 to 18 years of age. Children were defined as persons aged less than 14 years, adults were at least 14 years old.

### Randomisation and blinding

We used a cluster randomisation with the households serving as clusters. We prepared lists of random numbers with Microsoft Excel 2003 (Mircosoft™ Cooperation, Seattle, USA) which were divided between the three intervention groups. Each participating physician received a list of random numbers with the interventions represented in a 1:1:1 ratio. Eligible index patients were randomly assigned a number, which was then communicated to the study center. The resulting intervention was only communicated to the households with the physicians (as well as laboratory personnel) blinded from the randomisation results. Intervention material was given to the study sites in closed boxes marked only with the randomisation number. Recruiting physicians were not aware of the allocation of the numbers to the interventions and the boxes for the three intervention arms looked identical. After randomisation, participants were given their box by the physician's assistants.

### Interventions

The following three intervention groups were used: (i) Mask/Hygiene (MH) arm: households were provided with alcohol based hand-rub (Sterilium™, Bode Chemie, Germany) and surgical facemasks in two different sizes, one for children aged younger than 14 years (Child's Face Mask, Kimberly-Clark, USA) and one for adults (Aérokyn Masques, LCH Medical Products, France). If masks intended for participants younger than 14 years did not fit properly (as assessed by study personnel during the first household visit), we asked them to wear adult masks instead. Household also received information on the proper use of the interventions; (ii) Mask (M) arm: we provided the household with surgical facemasks and information on their correct use; (iii) Control (C) arm: no masks or hand rub was provided. All participating households received general written information on infection prevention [10]. Households received all necessary material (including a digital tympanic thermometer) on the day of recruitment and were called by study personnel immediately after leaving the study sites for instructions on how to use it correctly (provisional implementation of the intervention). Trained study personnel visited the household no later than 2 days after symptom onset of the index case. Using written information, study personnel demonstrated the correct use of the intervention material (full implementation of the intervention). We asked participants of the MH group to always use the provided hand rub after direct contact with the index patient (or other symptomatic household members), after having touched household items being used by the index patients and/or other symptomatic household contacts, as well as after coughing/sneezing, before meals, before preparing meals and when returning home. We asked all participants of the MH and M groups to wear masks at all times when the index patient and/or any other household member with respiratory symptoms were together in one room with healthy household members. Facemasks were to be changed regularly during the day and not to be worn during the night or outside the household.

### Follow up

The observation period for each household lasted 8 days, starting on the day of symptom onset of the index patient (day 1). We visited households on days 2, 3, 4, 6, and 8 (five times) or on days 3, 4, 6 and 8 (four times) depending on the day of recruitment. During these visits we obtained nasal wash specimens (or - if these were not possible - nasal swabs) from all participating household members. Antiviral medication was given to index patients and secondary cases by their individual physicians based on their clinical evaluation independent of study procedures. By definition, a "timely" antiviral therapy started within 2 days of symptom onset.

When household members developed fever (> 38.0°C), cough, or sore-throat they were asked to adopt the same preventive behaviour as the index patient (i.e. use facemasks or hand hygiene measures as required by index patients to protect other healthy household members) until the end of the observation period. All participants self-recorded symptoms (fever, shivering, measured temperature, cough, sore throat) and daily routines (incl. the time spent at home, and within close range (i.e. < 2 m) of the index patient) in a daily monitoring questionnaire.

### Outcome definitions

The primary outcome measure for secondary cases was qRT-PCR confirmed influenza infection. We defined a symptomatic secondary influenza virus infection as a laboratory confirmed influenza infection in a household member who developed fever (> 38.0°C), cough, or sore-throat during the observation period. We termed all other secondary cases as subclinical. A secondary outcome measure was the occurrence of ILI as defined by WHO [11] as fever plus cough or sore throat.

### Adherence

Participants of the M and MH groups also recorded daily adherence with facemasks, i.e. if they wore masks "always", "mostly", "sometimes", or "never" in the situation they had been asked to wear them. In the season 2010/11 they also recorded the number of masks used per day. Participants of the MH households additionally noted the number of hand disinfections per day. On day nine, study personnel conducted an exit questionnaire with all participants collecting information on (preventive) behaviour during the past 8 days, general attitudes towards NPI, the actual amount of used intervention materials and - if applicable - problems with wearing facemasks. We did not address potential problems with intensified hand hygiene. Parents answered the questionnaires on behalf of their children.

For the final analysis, two definitions of adherence were used, the first based on daily observations, the second on the behaviour of participants during the five consecutive days after implementation.

Used intervention material per household member was calculated by dividing the amount used per household by the number of household members.

### Reimbursement

Participants received a reimbursement of €150 for the large number of respiratory samples obtained.

### Sample collection and laboratory methods

For the collection of nasal wash, we used 5 mL of isotonic saline, which were instilled into one nostril with participants heads tilted backwards. Participants were asked to remain in this position for 10-15 s while making hard 'K' sounds without swallowing. Subsequently, the participants were told to tilt their heads forward and the fluid was collected in a sterile cup [12]. Nasal swabs were collected by using virus transport swabs (Mastaswab™; MAST Diagnostica, Reinfeld, Germany). Samples were stored refrigerated (at a temperature of approximately 5°C) before analysis [13]. Specimens were analysed by qRT-PCR at the Centre for Biological Security, Division of Highly-Pathogenic Viruses (season 2009/10) and the National Reference Centre for Influenza (season 2010/11) both part of the Robert Koch Institute in Berlin, Germany. RNA was extracted using either the MagNA Pure 96 DNA and Viral Nucleic Acid Small Volume Kit (Roche Applied Science, Mannheim, Germany) on MagNA Pure 96 instrument (Roche Applied Science) according to the manufacturer's instructions. Details about the PCR protocol as well as primer and probe sequences have been published elsewhere [14].

### Sample size estimation and statistical analysis

We assumed a SAR of laboratory confirmed infection of 20% in household contacts of the control group, based on data from another study of this group (SAR 26% [13]) as well as other published data on seasonal (18.4% [6]) and pandemic influenza (14.5% [15], 30% [16]). Assuming an average of 2 household contacts per household [13] and an intracluster correlation coefficient (ICC) of 0.3 [4-6], we estimated that 114 household members would be needed in each intervention arm to detect a 75% difference in secondary attack rates, i.e. 20% in the control group and 5% in the intervention groups, with 80% power and at a significance level of 5%.

Analysis was done by intention-to-treat. For descriptive analysis we used Student's *t*-test and Kruskal-Wallis one-way analysis of variance for numerical and chi-square tests for categorical variables. The intention-to-treat analysis was conducted in the following order:

1. Comparison of SAR between intervention groups via adjusted chi-square tests [17] (overall and stratified by virus subtype, season and time of implementation of intervention) to account for the cluster design of the study. We used a cluster bootstrapping technique for the calculation of 95% confidence intervals (95% CI) [18].

2. We used the generalized estimating equations (GEE) approach to fit logistic regression models [19] for evaluation and comparison of SAR between intervention groups. First, we calculated odds ratios (OR) for the outcome "laboratory confirmed influenza" with the following independent variables: (i) intervention group, (ii) intervention group (with pooled data of M and MH group), (iii) one separate model for each individual variable that may have influenced household transmission of influenza (i.e. age, sex, timely antiviral therapy of the index, vaccination of household contacts, etc.) adjusted for intervention group. This corresponds to a univariable analysis with the exception of the adjustment for intervention group.

3. Calculation of ORs for the clinical case definition (otherwise as in 2.).

4. Calculation of ORs for the outcome "laboratory confirmed influenza" to analyse the effect of the interventions while adjusting for variables with possible influence on influenza transmission. In a further model we used the variable intervention group with pooled data of M and MH group.

5. Calculation of ORs for the outcome "laboratory confirmed influenza" adjusting for variables with possible influence on influenza transmission in the following subgroups: (i) only data from season 2009/10, (ii) only data from season 2010/11, (iii) only data from households with full implementation of intervention < 36 h after symptom onset of the index case, (iv) only data from influenza A(H1N1)pdm09 cases.

We used a forced-entry method adjusting for variables potentially associated with risk of secondary infection. Sample sizes for these subgroup analyses were small and sometimes did not allow the inclusion of the full list of variables.

The per-protocol-analysis was conducted in the same way as the intention-to-treat analysis but only with data from participants who had followed the assigned interventions.

For all analyses, we used two-sided tests and considered p-values of < 0.05 as significant. We performed analyses with Stata software version 11 (Stata Corporation, Texas, USA).

### Ethics statement and trial registration

Ethics Committee approval was obtained from the institutional review board of Charité Universitätsmedizin Berlin. The study was registered with ClinicalTrials.gov (Identifier NCT00833885).

## Results

### Participants

**Figure 1:**
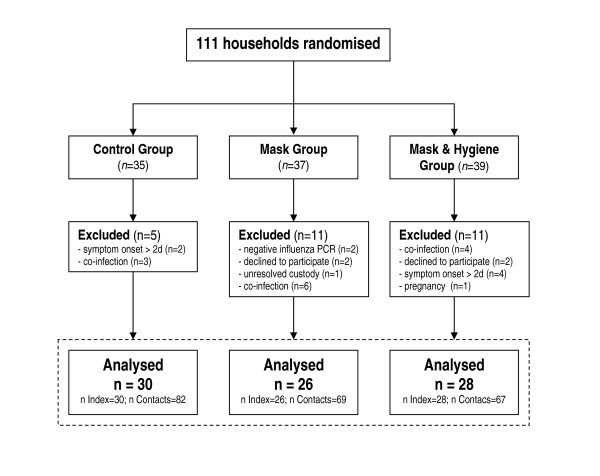
**Study flow diagram**.

Initially, we recruited 111 households which were randomised into one of the three intervention groups during the two study periods in 2009/10 and 2010/11 (Figure 1). After application of the exclusion criteria 30, 26 and 28 households remained in the Control-, M- and MH-groups for analysis. The total number of study participants was 302, of whom 84 were index patients and 218 household contacts. The study flow according to the CONSORT guidelines is shown in Figure 1.

Table 1 shows the baseline characteristics of index patients and household contacts of all analyzed households stratified by season of study participation and intervention group. One difference between the two study seasons was that in 2009/10 all viruses belonged to A (H1N1) pdm09, while in 2010/11 both A (H1N1)pdm09 as well as B viruses circulated (*p *= 0.14 for comparison within season). Another difference is that - compared to 2009/10 - the number of index patients receiving timely antiviral therapy was significantly higher in 2010/11 (*p *< 0.001). Furthermore, in 2010/11 a significantly larger number of both index patients (*p *= 0.045) and household contacts (*p *= 0.003) was vaccinated compared to 2009/10. Finally, in 2009/10 randomisation occurred significantly earlier after symptom onset compared to 2010/11 (*p *= 0.004) and a higher proportion of households was visited by study personnel within 36 h (*p *= 0.04). All other variables did not differ significantly between the two study seasons (Table 1).

**Table 1:** Baseline characteristics of index patients and household contacts, stratified by season and intervention group

Variable	2009/2010			2010/2011		
	
	Control Group	Mask Group	Mask & Hygiene Group	Control Group	Mask Group	Mask & Hygiene Group
**Index Cases - n**	**13**	**11**	**17**	**17**	**15**	**11**
Influenza virus subtype:						
A(H1N1)pdm09 - n/n (%)	13/13 (100)	11/11(100)	17/17 (100)	8/17 (47)	11/15 (73)	5/11 (45)
B - n/n (%)	0	0	0	10/17 (53)	4/15 (27)	6/11 (55)
Age (years) - median (IQR)	8 (7-10)	7 (5-10)	7 (4-10)	8 (6-11)	8 (4-9)	7 (5-9)
Age < 14 years - n/n (%)	12/13 (92)	11/11(100)	16/17 (94)	17/17 (100)	14/15 (93)	11/11 (100)
Sex male - n/n (%)	5/13 (39)	5/11 (45)	10/17 (59)	13/17 (77)	10/15 (67)	7/11 (64)
Vaccinated* - n/n (%)	0	0	0	2/17 (12)	2/15 (13)	0
Antiviral Therapy** - n/n (%)	3/13 (23)	0	2/17 (12)	11/17 (65)	11/15 (73)	10/11 (91)
Chronic Illness - n/n (%)	1/12 (8)	1/11 (9)	0	4/17 (24)	2/15 (13)	0
Symptoms:						
Fever/Chills - n/n (%)	13/13(100)	11/11(100)	17/17(100)	17/17(100)	15/15(100)	11/11(100)
Cough - n/n (%)	12/13 (92)	11/11(100)	16/17 (94)	16/17 (94)	14/15 (93)	10/11 (91)
Sore Throat - n/n (%)	4/13 (31)	6/11 (55)	9/17 (53)	10/17 (59)	9/15 (60)	7/11 (64)
Myalgia - n/n (%)	11/13 (85)	9/11 (82)	11/17 (65)	14/17 (82)	14/15 (93)	9/11 (82)
Symptom onset to rando-misation (hours) - mean ± SD	23.1 (3.7)	27.5 (12.4)	27.5 (11.5)	28.1 (13.9)	35.7 (8.7)	33.2 (11.2)
Symptom onset to first household visit - mean ± SD:						
< 36 h	6/13 (46)	5/11 (46)	9/17 (52)	4/17 (24)	1/15 (7)	1/11 (9)
36-48 h	4/13 (31)	2/11 (18)	4/17 (24)	9/17 (52)	7/15 (47)	6/55 (55)
48-60 h	3/13 (23)	4/11 (36)	4/17 (24)	0	2/15 (13)	1/11 (9)
> 60 h	0	0	0	4/17 (24)	5/15 (33)	3/11 (27)
Household size - mean ± SD	3.8 (1.2)	3.8 (1.2)	2.2 (1.1)	3.9 (1.3)	3.5 (0.9)	3.7 (1.1)

**Household Contacts - n**	**36**	**31**	**39**	**46**	**38**	**28**
Age (years) - median (IQR)	35 (18-40)	37 (12-43)	34 (19-42)	38 (12-43)	35(17-42)	35 (15-43)
Age < 14 years - n/n (%)	6/36 (17)	8/31 (26)	6/39 (15)	13/46 (28)	8/38 (21)	6/28 (21)
Sex male - n/n (%)	18/36 (50)	15/31 (49)	17/39 (44)	21/46 (46)	19/38 (50)	16/28 (57)
Vaccinated* - n/n (%)	0	1/31 (3)	1/39 (3)	6/46 (13)	7/38 (18)	1/28 (4)
Chronic Illness - n/n (%)	3/35 (9)	2/27 (7)	3/37 (8)	13/46 (28)	8/38 (21)	3/28 (11)

*IQR *Inter-Quartile-Range, *SD *Standard deviation. * Vaccination defined as vaccination against pandemic influenza in the pandemic season 2009/10 and trivalent seasonal vaccine in 2010/11, at least 14 days before symptom onset in index patient. ** Antiviral therapy defined as treatment with oseltamivir or zanamivir within 2 days of symptom onset

### Secondary attack rates (SAR)

Overall, there were 35 (16%) secondary cases of qRT-PCR confirmed influenza and 26 (12%) secondary ILI out of a total of 218 household contacts belonging to 84 households. Secondary laboratory confirmed cases occurred in 26 households, including 18 (69%) households with one secondary case, six (22%) with two secondary cases, and two (8%) with three secondary cases. When stratified by season, overall SAR was 13% (14/106) in 2009/2010 and 19% (21/112) in 2010/2011 (*p *= 0.3). For laboratory-confirmed cases SAR were not significantly lower in the M (9% (6/69)) and MH group (15% (10/67)) compared to the control group (23% (19/82); Table 2). In all stratified analyses (by influenza type, season, and implementation within 36 h after symptom onset) SAR of the M group was approximately reduced by 50% compared to the control group. In 2010/11 the MH group had markedly different SAR in A (H1N1) pdm09 households compared to B households. In A (H1N1) pdm09 households, SAR was highest in the control group and similarly low in the MH and M groups; but in B households it was highest in the MH group. When considering only households with early "full implementation" of the intervention (within 36 h after symptom onset), SAR of the MH group was substantially lower than in the control group.

**Table 2:** Secondary attack ratios of qRT-PCR confirmed influenza infection and clinical influenza, intention-to-treat analysis

Confirmation of influenza infection		Control Group		Mask Group		Mask & Hygiene Group		*p*-value^§^
		
		n/n	SAR (95% CI^#^)	n/n	SAR (95% CI^#^)	n/n	SAR (95% CI^#^)	
RT-PCR*	All cases	19/82	23 (13-35)	6/69	9 (3-19)	10/67	15 (5-27)	0.18
	
	Influenza A (H1N1) pdm09	13/56	23 (11-37)	6/58	10 (3-20)	4/50	8 (2-17)	0.16
	
	Influenza B	6/26	23 (5-48)	0/11	0	6/17	35 (7-60)	0.22
	
	Season 2009/2010	8/36	22 (10-35)	3/31	10 (0-26)	3/39	8 (0-18)	0.29
	
	Season 2010/2011	11/46	24 (9-42)	3/38	8 (0-19)	7/28	25 (4-45)	0.27
	
	Implementation of intervention **< 36 h **after symptom onset of index patient	5/22	23 (6-39)	1/14	7 (0-23)	1/24	4 (0-16)	0.17

Clinical (ILI**)	All cases	14/82	17 (7-29)	6/69	9 (3-17)	6/67	9 (3-16)	0.37
	
	Influenza A (H1N1) pdm09	10/56	18 (7-31)	6/58	10 (3-20)	3/50	6 (0-13)	0.29
	
	Influenza B	4/26	15 (0-39)	0/11	0	3/17	18 (0-37)	0.56
	
	Season 2009/2010	6/36	17 (6-31)	4/31	13 (3-30)	3/39	8 (0-16)	0.57
	
	Season 2010/2011	8/46	17 (2-35)	2/38	5 (0-14)	3/28	11 (0-25)	0.48
	
	Implementation of intervention **< 36 h **after symptom onset of index patient	4/22	18 (4-38)	2/14	14 (0-36)	1/24	4 (0-13)	0.41

*RT-PCR Real time polymerase chain reaction. **ILI Influenza-like-illness. ^§^p-values adjusted for cluster design of the study

^#^*95% CI *95% confidence interval. Confidence intervals were calculated using bootstrapping methods

SAR measured by the ILI case definition yielded somewhat lower results compared to those using the laboratory case definition, because not all laboratory-confirmed cases were also ILI cases.

Overall, differences in SAR were not significant, neither for laboratory confirmed secondary cases nor for ILI (Table 2), neither in primary analysis nor after stratification for season, influenza virus (sub)type or timing of the first household visit (Table 2).

### Multivariable and other analyses

In addition to the analysis of all three interventions groups, we also calculated odds ratios (OR) for secondary infection when intervention (i.e. M- and MH-group) groups were combined (Table 3). Although OR suggested a protective effect, this was not statistically significant. Among other individual variables with possible influence on secondary infection (such as sex, age, time spent at home, timely antiviral therapy of the index patient, and vaccination of the household contact) one variable stood out: Household members who spent at least 18 h of the day at home were significantly more likely to develop laboratory confirmed influenza infection (or ILI) compared to those who spent less time at home (Table 3).

**Table 3:** Separate models for predictors of qRT-PCR confirmed influenza infection and clinical influenza among included households.

Variable	RT-PCR confirmed Influenza			ILI		
	
	OR	95% CI	*p*-value	OR	95% CI	*p*-value
**Intervention Group**						
Control Group	Ref.			Ref.		
Mask Group	0.39	0.13-1.19	0.10	0.56	0.18-1.68	0.3
Mask & Hygiene Group	0.61	0.23-1.66	0.34	0.47	0.15-1.49	0.2
**Intervention Groups combined**						
Control Group	Ref.			Ref.		
Mask Group + Mask & Hygiene Group	0.50	0.21-1.20	0.12	0.51	0.2-1.28	0.15
Sex*						
Female	Ref.			Ref.		
Male	1.2	0.62-2.28	0.52	0.65	0.3-1.42	0.28
Age*						
Adult (> 14 yrs)	Ref.			Ref.		
Child	1.52	0.71-3.23	0.28	2.22	0.97-5.08	0.06
Time spent at home*						
< 18 hours/day	Ref.			Ref.		
≥18 hours/day	3.1	1.3-7.33	0.01	3.98	1.34-11.77	0.01
Therapy of Index patient*						
No timely therapy	Ref.			Ref.		
Timely therapy	1.63	0.70-3.81	0.26	1.07	0.42-2.7	0.89
Vaccination of Contact*						
No vaccination	Ref.			Ref.		
Vaccination	0.62	0.11-3.56	0.6	0.48	0.06-3.94	0.49

Independent variables: (i) intervention group, (ii) intervention group (with pooled data of Mask- and Mask&Hygiene group), (iii) one separate model for each individual variable that may influence household transmission of influenza (i.e. age, sex, timely antiviral therapy of the index, vaccination of household contacts) adjusted for intervention group

*OR *Odds ratio, *95% CI *95% Confidence Interval

*Models also included intervention group as independent variable

In a subgroup analysis, we examined the effect of the interventions only in households with early full implementation, and secondly only in households with (pandemic) A (H1N1) pdm09 infection. The first analysis was carried out in a subset of households where the intervention had been implemented no later than 36 h after symptom onset of the index patient. In this subset of 60 household contacts, we found a borderline significant protective effect of the MH intervention against laboratory confirmed influenza infection compared with the control group after adjustment for potential confounders (adjusted OR = 0.13; 95% CI = 0.01-1.28; *p *= 0.08; Table 4). After pooling the M and MH group, this effect became statistically significant (adjusted OR = 0.16; 95% CI = 0.03-0.92; *p *= 0.04). Among households with index cases infected with A (H1N1)pdm09 (162 household contacts) OR for a secondary laboratory confirmed infection were significantly lower in the MH group alone as well as the combined M- & MH-groups. Results of subgroup analyses using ILI as outcome were comparable to those using laboratory confirmed cases, but none were statistically significant.

**Table 4:** Multivariable analysis for predictors of qRT-PCR confirmed influenza infection and clinical influenza among included households in separate models allowing for within household correlation.

	Variable	RT-PCR confirmed Influenza			ILI		
		
		OR	95% CI	*p*-value	OR	95% CI	*p*-value
**Complete** **Data**	Intervention Group*						
	Control Group	Ref.			Ref.		
	Mask Group	0.39	0.13-1.17	0.09	0.61	0.2-1.87	0.39
	Mask & Hygiene Group	0.62	0.23-1.65	0.34	0.50	0.16-1.58	0.24
	Intervention Groups combined*						
	Control Group	Ref.			Ref.		
	Mask Group + Mask & Hygiene Group	0.50	0.21-1.19	0.12	0.55	0.22-1.40	0.21
**2009/2010**	Intervention Group*						
	Control Group	Ref.			Ref.		
	Mask Group	0.34	0.07-1.76	0.20	0.74	0.17-3.2	0.68
	Mask & Hygiene Group	0.23	0.05-1.13	0.07	0.41	0.09-1.84	0.24
	Intervention Groups combined*						
	Control Group	Ref.			Ref.		
	Mask Group + Mask & Hygiene Group	0.28	0.08-1.05	0.06	0.54	0.16-1.85	0.32
**2010/2011**	Intervention Group*						
	Control Group	Ref.			Ref.		
	Mask Group	0.35	0.07-1.63	0.18	0.37	0.06-2.35	0.3
	Mask & Hygiene Group	1.0	0.26-3.78	1.0	0.55	0.09-3.44	0.5
	Intervention Groups combined*						
	Control Group	Ref.			Ref.		
	Mask & Hygiene Group	0.63	0.19-2.11	0.45	0.45***	0.1-2.0	0.3
**Implementation** **of intervention** **< 36 h after** **symptom onset**	Intervention Group**						
	Control Group	Ref.			Ref.		
	Mask Group	0.21	0.02-2.02	0.18	0.63	0.08-4.92	0.66
	Mask & Hygiene Group	0.13	0.01-1.28	0.08	0.17	0.01-2.03	0.16
	Intervention Groups combined**						
	Control Group	Ref.			Ref.		
	Mask Group + Mask & Hygiene Group	0.16	0.03-0.92	**0.04**	0.34	0.06-2.13	0.25
**Only A/H1N1 pdm09 Infection**	Intervention Group*						
	Control Group	Ref.			Ref.		
	Mask Group	0.37	0.12-1.19	0.10	0.55	0.17-1.84	0.33
	Mask & Hygiene Group	0.27	0.07-0.99	**0.049**	0.26	0.06-1.15	0.08
	Intervention Groups combined*						
	Control Group	Ref.			Ref.		
	Mask Group + Mask & Hygiene Group	0.33	0.12-0.88	**0.027**	0.41	0.14-1.19	0.1

Intention-to-treat analysis. (n = 216 household contacts)

*OR *Odds ratio, *95% CI *95% Confidence Interval. * adjusted for age, sex, timely therapy of the index, vaccination of household contacts, time spent at home. ** adjusted for age, sex, timely therapy of the index, time spent at home. *** adjusted for age and sex

Considering that not all study participants followed the intended intervention in the group that they were assigned to, we conducted a (per-protocol) analysis among all participants who fully adhered to the study protocol. Because we implemented two interventions (facemask use and intensified hand hygiene) it was possible that one person adhered to one intervention, but not to the other. We therefore considered only adherence to facemask use for this analysis. We excluded participants of the control group when they wore a facemask during the study period, and we excluded participants from the M and MH group when they reported not wearing masks at all throughout the study. Apart from these exclusions of non-adherent study participants, we conducted the same analyses as for the intention-to-treat analysis. All OR of the M and MH group were below 1, mostly between 0.2 and 0.3 relating to a protective effect of 70%-80% for the interventions (Table 5). Significant results were reached in the M group when analysing the complete data set and in the M as well as the MH group when considering only A(H1N1)pdm09 households.

**Table 5:** Multivariable analysis for predictors of qRT-PCR confirmed influenza infection and clinical influenza among included households in separate models allowing for within household correlation.

	Variable	RT-PCR confirmed Influenza			ILI		
		
		OR	95% CI	*p*-value	OR	95% CI	*p*-value
**Complete** **Data**	Intervention Group*						
	Control Group	Ref.			Ref.		
	Mask Group	0.3	0.1-0.94	**0.04**	0.5	0.2-1.6	0.3
	Mask & Hygiene Group	0.59	0.2-1.5	0.3	0.49	0.2-1.6	0.2
	Intervention Groups combined*						
	Control Group	Ref.			Ref.		
	Mask Group + Mask & Hygiene Group	0.45	0.2-1.1	0.07	0.5	0.2-1.3	0.2
**2009/2010**	Intervention Group*						
	Control Group	Ref.			Ref.		
	Mask Group	0.21	0.03-1.4	0.1	0.7	0.13-3.4	0.6
	Mask & Hygiene Group	0.21	0.04-1.09	0.06	0.5	0.09-2.2	0.3
	Intervention Groups combined*						
	Control Group	Ref.			Ref.		
	Mask Group + Mask & Hygiene Group	0.21	0.05-0.9	**0.04**	0.53	0.14-2.08	0.4
**2010/2011**	Intervention Group*						
	Control Group	Ref.			Ref.		
	Mask Group	0.32	0.07-1.49	0.15	0.4	0.06-2.3	0.3
	Mask & Hygiene Group	0.94	0.25-3.5	0.9	0.51	0.09-3.3	0.5
	Intervention Groups combined*						
	Control Group	Ref.			Ref.		
	Mask Group + Mask & Hygiene Group	0.6	0.2-2.0	0.4	0.4	0.1-1.9	0.3
**Implementation** **of intervention** **< 36 h after** **symptom onset**	Intervention Group**						
	Control Group	Ref.			Ref.		
	Mask Group	0.23	0.02-3.02	0.26	0.7	0.07-7.9	0.8
	Mask & Hygiene Group	0.21	0.02-2.33	0.2	0.2	0.01-3.4	0.3
	Intervention Groups combined**						
	Control Group	Ref.			Ref.		
	Mask Group + Mask & Hygiene Group	0.22	0.03-1.6	0.1	0.4	0.05-3.3	0.4
**Only A/H1N1 pdm09 Infection**	Intervention Group*						
	Control Group	Ref.			Ref.		
	Mask Group	0.28	0.08-0.97	**0.04**	0.46	0.1-1.7	0.2
	Mask & Hygiene Group	0.26	0.07-0.93	**0.04**	0.26	0.06-1.17	0.08
	Intervention Groups combined*						
	Control Group	Ref.			Ref.		
	Mask Group + Mask & Hygiene Group	0.27	0.1-0.76	**0.01**	0.36	0.1-1.1	0.08

Per-protocol analysis. (n = 216 household contacts)

*OR *Odds ratio, *95% CI *95% Confidence Interval. * adjusted for age, sex, timely therapy of the index, vaccination of household contacts, time spent at home. ** adjusted for age, sex, timely therapy of the index, time spent at home

### Behaviour and adherence

The amount of remaining intervention material was assessed at the end of the study periods. Participants in the M group used a median of 12.9 (interquartile range [IQR]: 9.5-16) facemasks per individual, members of the MH group used a median of 12.6 (IQR: 7.8-14). There was no statistically significant difference between the seasons (data not shown). Only in 2010/11 did we assess the mean number of facemasks used per day and individual: participants in the M group used a mean number of 1.8 (SD: 1.8) and those in the MH group 1.7 (SD: 2.0) facemasks per day. The mean amount of alcohol based hand rub used over the study period by individuals in the MH group was higher in 2009/10 compared to 2010/11 (85.2 ml vs. 42.7 ml, *p *= 0.2).

#### Facemasks

**Figure 2:**
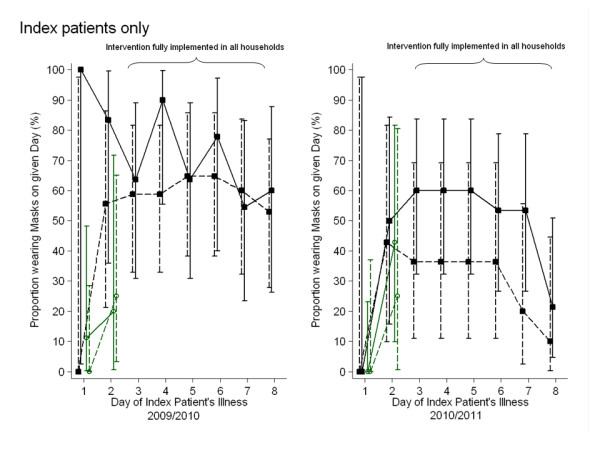
**Facemask adherence by index patients**. Daily proportion and 95% confidence interval of index patients wearing a facemask 'always' or 'mostly' in transmission-prone situations, in households assigned to wearing facemasks and practising intensified hand hygiene (MH group; dashed line) or to only wearing facemasks (M group; continuous line), stratified by season. Symbols represent the proportion of participants wearing facemasks before (green, hollow circles) and after (black squares) the intervention was fully implemented in the household.

**Figure 3:**
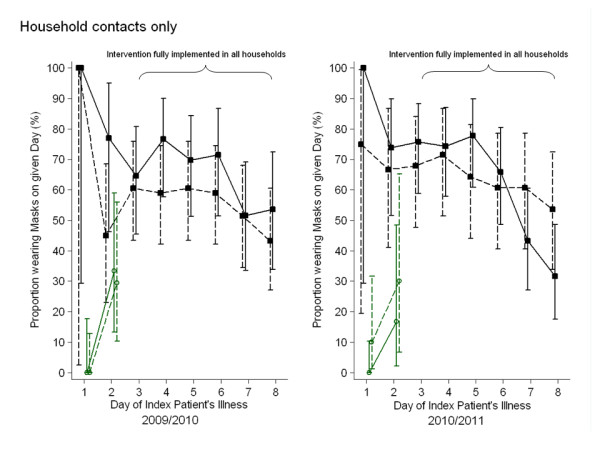
**Facemask adherence by household contacts**. Daily proportion and 95% confidence interval of household contacts wearing a facemask 'always' or 'mostly' in transmission-prone situations, in households assigned to wearing facemasks and practising intensified hand hygiene (MH group; dashed line) or only wearing facemasks (M group; continuous line), stratified by season. Symbols represent the proportion of participants wearing facemasks before (green, hollow circles) and after (black squares) the intervention was fully implemented in the household.

We used two definitions to describe adherence to wearing masks. The first evaluated daily adherence and considered a participant as "adherent" if they wore a mask "always" or "mostly" on each day as required by the study protocol (adherence definition 1). The second definition evaluated behaviour of participants during the first 5 days after implementation of the intervention [6]. A participant was termed adherent if they wore a facemask "always" or "mostly" on each of the first 5 days after full implementation of the intervention (adherence definition 2). Figures 2 and 3 display the data for adherence (according to definition 1) to facemask use in the M and MH group separately for index patients (Figure 2) and household contacts (Figure 3).

In general, daily adherence was good, reaching a plateau of over 50% in nearly all groups (M and MH groups; 2009/10 and 2010/11) from the third day on (by then the intervention had been implemented in all households). A gradual decline towards lower adherence began around the sixth day of the index patient's illness. A further observation was that in 2010/11 MH index patients were less adherent than M index patients (Figure 2, right panel; difference not statistically significant) while the two groups were fairly similar during the 2009/10 pandemic. Similar differences can be observed when only the first 5 days following full study implementation were considered (definition 2) (Table 6). Adherence behaviour of household contacts was similar in both seasons.

**Table 6:** Adherence to interventions in index patients and household contacts by intervention group and study season

Variables	Index				Contacts			
	
	Mask Group		Mask & Hygiene Group		Mask Group		Mask & Hygiene Group	
	
	2009/ 2010	2010/ 2011	2009/ 2010	2010/ 2011	2009/ 2010	2010/ 2011	2009/ 2010	2010/ 2011
**Hygiene variables**								

Number of hand disinfections per day - mean ± SD	NA		7.4 (6.3-8.5)*	4.1 (3.3-4.8)*	NA		8.8 (7.9-9.6)	7.5 (6.5-8.5)
Adherent to intensified hand hygiene during each of the five days after implementation of the intervention (adherence def. 2) - n/n (%)**	NA		8/17 (47)*	1/9 (9)*	NA		15/39 (38)	13/28 (46)
Hand disinfection/-washing after coming home*** - n/n (%)	7/9 (78)	7/14 (50)	10/16 (63)	6/11 (55)	11/23^#^ (64)	21/30^#^ (70)	28/32^#^ (88)	20/22^#^ (91)
Hand disinfection/-washing after touching contaminated objects*** - n/n (%)	1/11 (9)	0	3/17 (18)	1/11 (9)	11/23^#^ (48)	16/30^#^ (53)	28/32^#^ (88)	16/22^#^ (73)
Hand disinfection/-washing before eating*** - n/n (%)	8/11 (73)	6/15 (40)	13/17 (77)	6/11 (55)	23/23^#^ (100)	27/30^#^ (90)	28/32^#^ (88)	15/22^#^ (68)
Hand disinfection/-washing after coughing/sneezing*** - n/n (%)	5/11 (46)	0	9/17 (53)	3/11 (27)	17/30^#^ (57)	16/38^#^ (42)	22/38^#^ (58)	8/28^#^ (29)

**Mask variables**								

Adherent to facemask use during each of the five days after full implementation of the intervention (adherence definition 2) - n/n (%)^##^	6/11 (55)	7/15 (47)	7/17 (41)	2/11 (18)	17/31 (55)	18/39 (46)	17/38 (45)	13/28 (46)
Wore facemask when being in the same room with index/contact*** - n/n (%)	8/10 (80)	8/15 (53)	13/16 (81)	6/11 (55)	23/31 (74)	32/37 (87)	26/38 (68)	24/28 (86)
Wore facemask when being in close contact to index/contact*** - n/n (%)	7/10 (70)	9/15 (60)	13/16 (81)	5/11 (46)	24/31 (77)	34/38 (90)	27/38 (71)	21/28 (75)
Wore facemask when providing care to index/being provided for by household contact*** - n/n(%)	6/9 (67)	4/5 (80)	6/12 (50)	3/7 (43)	10/16 (63)	13/16 (81)	12/21 (57)	12/13 (92)

*NA *not applicable, *HD *hand disinfection, *SD *standard deviation. * *p *< 0.05 between seasons. ** **Adherence to hand hygiene measure **was defined as disinfection of hands at least 5 times per day during the 5 day after full implementation of the intervention (based on data from daily questionnaire). *** based on data from exit questionnaire. #only adults. ## **Adherence to facemask intervention **was defined as wearing a facemask "mostly" or "always" on each of the 5 days after full implementation of the intervention (based on data from daily questionnaire)

In both season, the majority of participants (107/172, 62%) did not report any problems with mask wearing. This proportion was significantly higher in the group of adults (71/100, 71%) compared to the group of children (36/72, 50%) (*p *= 0.005). The main problem stated by participants (adults as well as children) was "heat/humidity" (18/34, 53% of children; 10/29, 35% of adults) (*p *= 0.1), followed by "pain" and "shortness of breath" when wearing a facemask.

#### Hand hygiene

**Figure 4:**
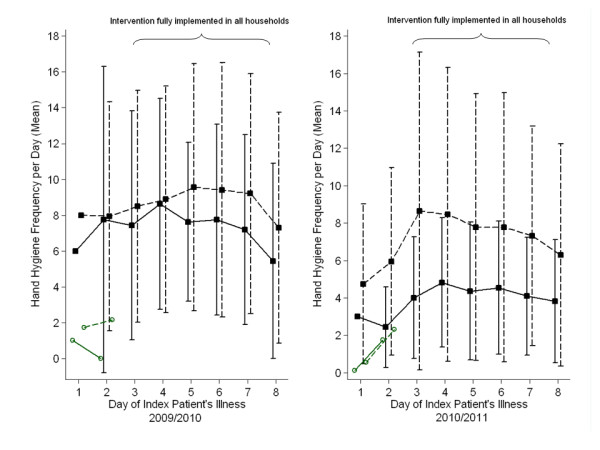
**Adherence to hand hygiene measures by index patients and household contacts**. Frequency of daily hand disinfection (mean, standard deviation) in participants assigned to the MH group, stratified by season. Symbols represent the mean frequency of hand disinfection before (green, hollow circles) and after (black squares) the intervention was fully implemented in the households. Data of index patients are depicted by a continuous line, data of household contacts by a dashed line.

For the daily evaluation of adherence to hand hygiene measures we used the number of hand disinfections as indicated by MH participants (adherence definition 1). A MH participant was termed adherent according to definition 2 if they had disinfected their hands at least five times per day on each of the 5 days after full implementation of the intervention. Similar to the low facemask adherence of MH index patients in 2010/11, this group also displayed low hand hygiene adherence compared to the index patients of 2009/10. As in facemask adherence, household contacts kept a relatively stable level of adherence in both seasons (Figure 4). Also the 5-day adherence (adherence definition 2) of index patients of the MH group dropped from 41% in 2009/10 to 18% in 2010/11 (*p *= 0.2) while it did not drop in household contacts (Table 6).

We validated reported data on adherence by comparing the indicated number of daily mask use and number of disinfections with the remaining intervention material in each household at the end of the study period. For both interventions, cumulative subjective information by participants correlated well with the objective measurements of the remaining material (facemasks: r = 0.64, *p *< 0.001 (only possible for season 2010/11 as information on the number of facemasks used per day was only collected in the season 2010/11); hand hygiene: r = 0.53, *p *< 0.001).

We observed "contamination" between intervention groups in two control households only in the season 2009/10, one reported wearing masks, the other reported wearing masks and using alcohol based hand sanitizer.

Examination of further potentially relevant behavioural variables, such as daily time spent at home during the study period, time spent at close range of the index patient, sleeping in the same room or taking meals with the index patient, did not result in significant differences between the study groups or between influenza seasons (data not shown).

## Discussion

We present results of a cluster randomised trial on the effectiveness of facemasks and hand hygiene in preventing household transmission of influenza. The trial was conducted in Berlin during the first two seasons after the onset of the influenza A (H1N1)pdm09 pandemic.

In primary intention-to-treat analysis of all data, the interventions did not lead to statistically significant reductions of SAR in household contacts. However, in a secondary analysis among households with full implementation of the intervention within 36 h after symptom onset, the combined participants from M and MH groups had a significantly lower chance of influenza infection compared to controls. With one exception (MH households in 2010/11), we observed a non-significant, but consistent and substantial reduction of the OR for influenza infection in both intervention groups (M, MH) and for both case definitions (laboratory confirmed and clinical).

A per-protocol analysis showed comparable results of lowered influenza transmission in the intervention groups which were statistically significant in the M group when analysing the complete dataset, and - among A (H1N1) pdm09 households - in the combined analysis of the M and MH groups. The main drawback of the study was that we did not reach the number of households that we had aimed and planned for, one of the reasons being the at best moderate influenza season 2010/11. Our sample size calculation was based on a 75% reduction of risk due to the interventions. This may seem questionably high in comparison to other studies, however based on experience from our pilot study we felt that adherence would be better than reported in the Hong Kong [5] and Bangkok [7] studies. We therefore expected a larger effect size in our main study.

The reason for the high SAR of 25% in MH households from the 2010/11 season (with 35% the SAR was even higher when only households with Influenza-B positive index patients were considered) remains unclear. However, we hypothesize that the particularly low adherence of MH index patients to both interventions during the 2010/11 season might have influenced this observation.

The fact that we observed a significant effect of the combined M and MH intervention only after restriction of analysis to households with early (< 36 h after symptom onset) implementation of the interventions is in agreement with Cowling et al. who had investigated a hand hygiene intervention as well as hand hygiene plus facemask use [5]. The importance of early implementation of any intervention is plausible given high levels of viral shedding during the initial period of influenza infection [20] as well as the short incubation period [21]. Recently, Donnelly et al. quantified the probability of a transmission event by an infectious person relative to the onset of symptoms [22] and showed that peak transmission occurred on days 1, 2 and 3 of the infectious patient's illness. Merely 18% of transmission events took place more than two days after symptom onset of the index patient.

An Australian cluster-randomized household study conducted in a pre-pandemic winter season investigated the effect of the use of facemasks (surgical, or N95) on the risk of respiratory infections with all index cases being children and having an influenza-like illness of any, even unknown, etiology [6]. Intention-to-treat analysis did not yield significant results, however, good adherence to facemask use proved to be significantly protective in a per-protocol analysis.

Two further cluster-randomized household studies failed to see any significant effects of intervention measures (facemasks or hand hygiene) even in secondary analyses. A French study investigated the efficacy of facemasks in the pre-pandemic influenza season 2008/09 [4]. Although a planned second season was not followed through due to the onset of the influenza A (H1N1)pdm09 pandemic, reported SAR after the first season were quite similar in intervention and control groups. Adherence was reported to be good, but only a clinical case definition (ILI) was used for secondary cases, thus probably missing a- and oligosymptomatic secondary cases. Only index patients were supposed to wear the masks, and mean age of index patients in the intervention arm was 25 years. Since young children and infants may play a more important role in the (household) transmission of influenza [23-25], it is possible that these factors may have led to a cumulative underestimation of the real effect of facemasks.

The second study failing to see an effect of NPI in the household setting comes from Bangkok, Thailand and was conducted between April 2008 and August 2009 [7]. Interventions tested were facemasks combined with handwashing, and handwashing alone. Although study size was large corresponding to high statistical power, the fact that 90% of index patients slept in the same bedroom as their parents without wearing facemasks during the night may have overcome any protective effect conferred by the interventions during daytime. In addition, authors describe a considerable amount of contamination between intervention groups, which may have further concealed true effects of the interventions.

In our study adherence to both interventions was good. After full implementation of the interventions approximately 50% of M and MH participants (index and contacts) wore facemasks "mostly" or "always" during the daytime in situations as required by the study protocol, and MH participants disinfected their hands approximately 7-8 times per day; only MH index cases in 2010/11 had lower adherence values for both interventions. Comparison with other studies is difficult for a number of reasons, particularly because interventions differed. Cowling [5] defined facemask adherence similar to the present study and reported similar facemask adherence in index cases (49%), but worse in household contacts (26%). Adherence to wearing facemasks in the Canini [4] study can be compared to our study for 2010/11, because the number of facemasks used per day was measured only in this season. The results were comparable in both studies.

Hand hygiene was part of the trial design only in the Simmermann [7] and Cowling studies [5]. MH index patients in our study disinfected their hands between 4.1 (2010/11) and 7.4 times (2009/10) per day, the index patients in the Simmerman study washed their hands 4.1 times per day. Adherence to hand disinfection by index patients over the course of the study (adherence definition 2) ranged between 9% (2010/11) and 47% (2009/10) in our trial, compared to Cowling et al. with 33% and 36% in the two groups assigned hand hygiene interventions. Among household contacts in the MH group of our study adherence was higher in every parameter measured compared to Simmerman et al. and Cowling et al.. Considering that facemask and perhaps also disinfectant use in household settings may be much less accepted in European compared to Asian countries [26], the high overall adherence of both interventions in our study is remarkable. However, adherence data in all studies were based on self reporting and differences in reporting behaviour may have influenced results.

Compared to 2010/11 adherence may have been higher during the pandemic season 2009/10, but differences were not statistically significant. Increased use of or willingness to use preventive measures, such as facemasks or hand hygiene, was also documented during the SARS epidemic as well as during the pandemic influenza A (H1N1)pdm09 [27-29]. As we reported previously [9], adherence was good in adults and children alike, and although difficulties with facemasks were more frequently reported by children compared to adults, the numbers were not high. One notable exception with considerably lower adherence in 2010/11 compared to 2009/10 was observed in index patients of the MH group. Because physical interventions used by infected children may have the largest effect on the reduction of spread [30] and most index cases were children, it is possible that their reduced adherence has negatively affected transmission rates in MH households resulting in higher SAR in this intervention group in 2010/11.

In general, we believe that our data for adherence and tolerability would support a recommendation to use non-pharmaceutical interventions in a pandemic.

Several limitations may have influenced the results of this study. As in all previous studies on this subject, our study design resulted in delays between symptom onset of the index patients and implementation of the intervention. This delay could be as long as 3 days in some households during the 2010/11 season. Although we tried to address this problem by calling the households for preliminary instructions directly after enrolment at their physicians' office, this does not substitute for a personal visit with a demonstration of the intervention in the household. This may have led to an underestimation of the true effect of the interventions.

A further limitation of our study is that we cannot determine whether a possible protective effect of wearing facemasks is more attributable to their use by index patients or by household contacts (or both), nor can we say if intensified hand hygiene provides any additional protection. Regarding the first question, there are data from a Dutch experimental study suggesting that the use of masks may be more effective for inward than for outward protection which would favour the importance of healthy persons wearing them [31]. This is in line with the results of the French trial [4] which stated in its protocol that facemasks were only to be worn by index patients and which could not show any significant protective effect in this setting. Regarding the role of hand hygiene, existing data from clinical trials are inconclusive. The study from Thailand found no effect, neither for facemasks nor for hand hygiene [7]. In the analysis of households where the intervention was applied within 36 h the Hong Kong study saw a (non-significant) effect of hand hygiene alone which became stronger and significant in the MH arm [5]. The investigators of a study among university students observed comparable reductions in ILI both in the facemask only as well as the facemasks plus hand hygiene groups suggesting that the addition of a hand sanitizer did not increase the effect of facemasks, or at least not substantially [3]. Nevertheless, a recent Cochrane review on the subject came to the conclusion that hand hygiene is generally effective in reducing the spread of respiratory viruses [30].

A further limitation is the fact that laboratory testing of household contacts was only conducted for the virus subtype the index patient was infected with. This could have led to an underestimation of secondary cases.

Finally, we cannot rule out the possibility that behaviour of participating households may have been influenced by monetary incentives and frequent household visits. However, they did not differ in all three study arms so we do not expect this to have biased our results. Furthermore, the other clinical trials had a similar design so that it should not endanger comparability of results.

The strengths of this study include laboratory confirmation of primary and secondary cases with qRT-PCR, the serial testing of all household members over the study period irrespective of respiratory symptoms, and the low degree of contamination between the intervention groups.

## Conclusions

In conclusion, results of our study contribute to the body of evidence that NPI may be effective in preventing transmission of influenza in households. Prerequisites include early implementation of the intervention and good adherence.

We were also able to show that the use of facemasks in particular is tolerable and acceptable for adults and children alike, both as household contacts and index cases, highlighting the fact that these measures could play an important role in the interruption of influenza transmission within households. Future research should focus on the differential importance of facemask use by index cases or household contacts as well as the independent role of hand hygiene in the prevention of influenza transmission.

## Competing interests

The authors declare that they have no competing interests.

## Authors' contributions

TS designed the study, co-ordinated the execution, executed the study, performed the statistical analysis, interpreted the results, and wrote the manuscript. UB developed the study idea, designed the study, supervised its co-ordination, data analysis and interpretation, and wrote the manuscript. CR designed the study, co-ordinated the execution, executed the study, and wrote the manuscript. SBS executed the study, and wrote the manuscript. IK analysed the data and wrote the manuscript. WH designed the study, and wrote the manuscript. GK provided important intellectual contributions to design, coordination and analysis of the study, and wrote the manuscript. BS, AN, KS, JD, and JM carried out the laboratory analysis, interpreted data, and wrote the manuscript. All authors read and approved the final manuscript.

## Pre-publication history

The pre-publication history for this paper can be accessed here:

http://www.biomedcentral.com/1471-2334/12/26/prepub
